# Deriving site-specific soil clean-up values for metals and metalloids: Rationale for including protection of soil microbial processes

**DOI:** 10.1002/ieam.1513

**Published:** 2014-02-26

**Authors:** Roman G Kuperman, Steven D Siciliano, Jörg Römbke, Koen Oorts

**Affiliations:** † US Army Edgewood Chemical Biological Center, Aberdeen Proving GroundMaryland; ‡ Department of Soil Science and Toxicology Program, University of SaskatchewanSaskatoon, Saskatchewan, Canada; § ECT Oekotoxikologie GmbHFloersheim, Germany; ‖ Arche (Assessing Risks of Chemicals)Ghent, Belgium

**Keywords:** Metal, Microbial processes, Risk assessment, Soil, Toxicity

## Abstract

Although it is widely recognized that microorganisms are essential for sustaining soil fertility, structure, nutrient cycling, groundwater purification, and other soil functions, soil microbial toxicity data were excluded from the derivation of Ecological Soil Screening Levels (Eco-SSL) in the United States. Among the reasons for such exclusion were claims that microbial toxicity tests were too difficult to interpret because of the high variability of microbial responses, uncertainty regarding the relevance of the various endpoints, and functional redundancy. Since the release of the first draft of the Eco-SSL Guidance document by the US Environmental Protection Agency in 2003, soil microbial toxicity testing and its use in ecological risk assessments have substantially improved. A wide range of standardized and nonstandardized methods became available for testing chemical toxicity to microbial functions in soil. Regulatory frameworks in the European Union and Australia have successfully incorporated microbial toxicity data into the derivation of soil threshold concentrations for ecological risk assessments. This article provides the 3-part rationale for including soil microbial processes in the development of soil clean-up values (SCVs): 1) presenting a brief overview of relevant test methods for assessing microbial functions in soil, 2) examining data sets for Cu, Ni, Zn, and Mo that incorporated soil microbial toxicity data into regulatory frameworks, and 3) offering recommendations on how to integrate the best available science into the method development for deriving site-specific SCVs that account for bioavailability of metals and metalloids in soil. Although the primary focus of this article is on the development of the approach for deriving SCVs for metals and metalloids in the United States, the recommendations provided in this article may also be applicable in other jurisdictions that aim at developing ecological soil threshold values for protection of microbial processes in contaminated soils.

## INTRODUCTION

Microorganisms are critical to terrestrial biogeochemical cycles and are key to sustaining the functioning of soil ecosystems (Supplemental Data Table S1). Microorganisms significantly contribute to soil fertility through nutrient cycling and the decomposition of organic matter. Scientists (Jaensch et al. 2007; Smolders et al. 2009; Swartjes 2011; Faber and van Wensem 2012) and regulatory agencies (EC 2002; UKEA 2002; CCME 2006; ECHA 2008a; NEPC 2011) acknowledge the ecological relevance of soil microbes. Therefore, the determination of effects on microbial processes is an integral part of many environmental risk assessments of chemicals in soil. Furthermore, many national and international jurisdictions that have developed threshold concentrations (e.g., clean-up levels) for contaminants in soil include microbial responses in their ecological risk assessment framework (ECHA 2008a; NEPC 2011).

The US Environmental Protection Agency (USEPA) derived ecological soil screening levels (Eco-SSLs) (USEPA 2005) for 24 contaminants, including 17 metals, to aid in assessing risk posed to plants and animals at hazardous waste sites. As discussed by Kapustka (1999), soil microbial processes were not included in this derivation because microbial endpoints present exceptional difficulty in terms of interpreting the severity of responses to chemicals. Microbial population, community, and functional measurement endpoints change rapidly and across very short spatial scales. Measurement endpoints of microbes tend to be highly responsive to changes in temperature, moisture, O_2_, and many other noncontaminant factors. Functional redundancy across broad taxonomic groups enables large changes in community composition without remarkable changes in rates of functions or processes (e.g., decomposition, respiration, nitrification). The focus of terrestrial risk assessment was on protection of vegetation, invertebrates, and vertebrates. It was assumed that changes in microbial processes do not necessarily result in consequences to plant and animal populations or communities, and therefore, microbial functions should not be included. It was therefore concluded at that time by USEPA (2003) that 1) protecting higher-order organisms (plants, soil invertebrates, and wildlife) would also protect the microbial communities, 2) there is too much uncertainty in the actual significance and relevance of soil microbial toxicity test data to warrant inclusion in screening-level ecological risk assessment, and 3) it is exceedingly difficult to relate specific microbial activities with indications of adverse and unacceptable environmental conditions. Despite the decision to not incorporate microbial toxicity data into Eco-SSL derivation, it was recognized that the well-being of the soil microbial community needs to be included in ecological risk assessment (Chapman 1999; Hull et al. 1999).

Since the release of the first draft of Eco-SSL Guidance in 2003, multiple studies, including those discussed in this article, have generated data showing that the metal sensitivity of soil microbial processes is comparable to that of soil invertebrates and terrestrial plants (Giller et al. 2009). These data indicate that microbial communities are essential for sustaining functional soil ecosystems (e.g., microbial communities support plant growth and protect groundwater by degrading contaminants) and sustaining effective bioremediation technologies (Griffiths et al. 2000). The Eco-SSL Guidance also expressed concerns with uncertainties about the scale and dynamic nature of soil microbial communities and their functional processes; however, methods for assessing a variety of soil microbial endpoints are now standardized, thus addressing these concerns. This contribution to the special publication series is one of several articles generated from a workshop entitled “Ecological Soil Levels—Next Steps in the Development of Metal Clean-Up Values” held September 17–21, 2012, in Sundance, Utah (Wentsel and Fairbrother, this issue). Among its main objectives are to provide the rationale for inclusion of soil microbial processes in the development of soil clean-up values (SCVs) and ecological soil threshold values in general for metals and metalloids. This article discusses relevant test methods for assessing metal toxicity to microbial functions in soil. This article presents examples of case studies that incorporated microbial toxicity data into regulatory frameworks, then provides recommendations for the inclusion of microbial data in the derivation of SCVs and similar ecological soil threshold values, and will conclude with a brief overview of the remaining knowledge gaps that should be addressed in future research.

## TEST METHODS FOR SOIL MICROBIAL PROCESSES

Unlike tests with plants and invertebrates that assess the performance metrics of individual test species, toxicity tests with microorganisms are based on effects on the functional processes or the composition of the soil microbial community. Although tests with plants and invertebrates rely on a limited number of standard test species, microbial toxicity tests are based on the indigenous community living in the soil tested because the vast majority of these organisms cannot be cultured independently of the test media. For example, it is not possible to collect a soil sample, process it, spike it with a test solution, and then add the microbial community to be evaluated. Instead, the microbial community is inherent in the soil ecosystem. Soil microbial community composition is linked to soil properties such as pH (Fierer and Jackson 2006), fertility (Grayston et al. 2004; Allison et al. 2007), texture (Carson et al. 2010), and mineralogy (Reith et al. 2012), and it is also linked to biotic factors such as life history (Bissett et al. 2010) and indigenous plant species. Thus, soil parameters influence not only how a metal interacts with the organisms in standard toxicity tests but also impact the specific composition of the microbial community developed in the soil and how it responds to stress. For example, the nitrifying community response and adaptation to metal stress is dependent on the type of organisms present (Mertens et al. 2009) and the food source (Ruyters et al. 2010), and these 2 parameters are soil dependent (Ma et al. 2008). Thus, when one assesses the sensitivity of a soil microbial community across multiple soils, as has been done for Cu, Ni, Zn, and Mo, the assessment includes the influences of different soil parameters on the relative bioavailability of metals and the inherent biological resilience of microbial communities. Although soil properties also influence the composition and resilience of terrestrial plant and invertebrate communities formed in a soil, this is not reflected in the standard single-species toxicity tests with plants and invertebrates that are commonly used as a basis for derivation of soil threshold concentrations, unless these standard tests are modified for use with natural soils.

An additional difference between microbial-based tests and single-species tests with plant and invertebrate species is that the short generation time associated with microorganisms, such as bacteria, enables these organisms and associated processes to adapt to metal impact faster than plant or invertebrate species. Thus, tests that are of long duration may report that the rate of the microbial process tested has been unaffected, whereas the community composition has been significantly changed. Because of functional redundancy, individual species may indeed decline without an adverse effect on important functions. Such a situation would potentially indicate a negative effect if the resilience of the microbial community is affected, but would not be apparent if the test is only used to assess functional processes. It is not clear how resistance and resilience of microbial communities will respond to metal stress (Bissett et al. 2013). One study suggested that long-term metal exposure had little effect on resistance and resilience (Mertens et al. 2010). It is generally accepted that it is more relevant to protect the functions provided by soil microorganisms (e.g., respiration, mineralization, nitrification) than to protect individual microbial species.

### Standardized tests

During the last 30 years, various microbial test methods have been developed and standardized by international (e.g., the Organization of Economic Co-operation and Development [OECD] and the International Organization for Standardization [ISO]) and national organizations (USEPA 1987, 2008). In general, OECD publishes methods for investigating individual chemicals, whereas ISO focuses on methods to investigate chemical mixtures in environmental substrates such as wastewater or soils. Standard guidelines from both organizations have been used to study the effects of metals on soil microorganisms. Relevant methods include OECD and ISO standardized tests, those currently in the standardization process and several nonstandardized methods (Table1).

**Table 1:** Standard guidelines for the testing the effects of chemicals on microorganisms published by OECD and ISO

Number	Title	Year
OECD 216	Soil microorganisms, N transformation test	2000a
OECD 217	Soil microorganisms, C transformation test	2000b
OECD GD 56	Guidance document on the breakdown of organic matter in litterbags	2006
ISO 14238	Determination of N mineralization and nitrification in soils and the influence of chemical on these processes	1997
ISO 14240-1	Determination of soil microbial biomass–Part 1: Substrate-induced respiration method	1997a
ISO 14240-2	Determination of soil microbial biomass—Part 2: Fumigation-extraction method	1997b
ISO 15685	Determination of potential nitrification and inhibition of nitrification—Rapid test by ammonium oxidation	2004
ISO 16072	Laboratory methods for determination of microbial soil respiration	2002
ISO 17155	Determination of abundance and activity of soil microflora using respiration curves	2002
ISO 18311 (draft)	Method for testing effects of soil contaminants on the feeding activity of soil dwelling organisms—Bait-lamina test	2012
ISO 22939	Measurement of enzyme activity patterns in soil samples using fluorogenic substrates in micro-well plates	2010
ISO 23753-1	Determination of dehydrogenase activity in soils—Part 1: Method using TTC	2005
ISO 23753-2	Determination of dehydrogenase activity in soils—Part 2: Method using INT	2005
ISO 29843-1	Determination of soil microbial diversity—Part 1: Method by PLFA and PLEL analysis	2010
ISO 29843-2	Determination of soil microbial diversity—Part 2: Method by PLFA using the “simple PLFA extraction method”	2011

ISO = International Organization for Standardization; OECD = Organization of Economic Co-operation and Development; PLEL = phospholipid ether lipids; PLFA = phospholipid fatty acid analysis; INT = iodotetrazolium chloride; TTC = triphenyltetrazolium chloride.

Different versions of these tests have been used for decades in soil ecological studies to assess basic functions of microorganisms, especially those applicable to the C (i.e., respiration: OECD 217; ISO 16072) and N (i.e., OECD 216; ISO 14238) cycles. Carbon methods are probably used most often because their results can easily be recalculated to yield soil microbial biomass. However, because the C cycle has low sensitivity toward many chemicals, it was removed from the list of required tests for the registration of pesticides in the European Union (EU) (EFSA 2007). Almost as common, are tests addressing enzymes, either individually (e.g., dehydrogenase: ISO 23753-1; ISO 23753-2) or several simultaneously (ISO 22939). The latest developments in standardization focus on assessing the community composition of soil microorganisms using patterns of phospholipid fatty acids and phospholipid ether lipids as the main endpoints. These methods have often been used to determine the effects of metals on soil microorganisms, and they provide an estimate of the sensitivity to metal contamination of soil microbial community composition (Frostegard et al. 1996; Grayston et al. 2004).

### Nonstandardized tests

Numerous studies are published on nonstandard tests or adaptations of standard tests. As an example, potential nitrification rate (PNR) can be assessed using either a soil (Smolders et al. 2001) or a slurry assay (Schafer et al. 2007), or a stable isotope pool dilution assay (Ma et al. 2007). The standard approach for assessing nitrification rates in soil is the ^15^N isotope pool dilution test. For metals, the PNR test is usually run in solid state (Broos et al. 2005), which provides an estimate of metal toxicity in soil. The slurry assay can provide data to calculate the PNR (mg NO_2_-N kg fresh soil/day) from the linear increase of soil NO_2_-N after the addition of substrate into a slurry. Although some researchers questioned whether slurries can reflect in situ activity, Shafer et al. (2007) and Harvey et al. (2012) demonstrated that the slurry PNR test provides the same activity measures as the N^15^ isotope pool dilution test. The rationale for using the slurry test is that if a pollutant induces N immobilization, as can happen with hydrocarbons or other labile pollutants, it will confound the solid state PNR test. Therefore, we recommend using the solid state PNR for metals, which presumably do not induce immobilization, and the slurry PNR for chemicals that can induce N immobilization (such as hydrocarbons) to avoid confounding the solid-state PNR due to N immobilization.

Standard and nonstandard tests can both be used within the framework of effects assessment or risk assessment. In general, toxicity data generated from standardized tests, as prescribed by organizations such as OECD, ISO, and USEPA, will need less scrutiny than nonstandardized test data, which will require a more thorough check on their compliance with reliability criteria before being used. For some metals, nonstandardized tests have been used to prepare species sensitivity distributions (SSDs) (Carlon 2007; Smolders et al. 2009). Only toxicity data obtained in studies using organism exposures to contaminants in natural soil should be used because results of tests performed in other substrates (e.g., nutrient solution, agar, pure quartz sand, farmyard manure, or exposure in soil suspensions) are judged to be not representative of soils. The reliability criteria do not exclude enzyme assays, however, many enzyme assays are of low relevance for natural exposure conditions. The enzymatic activities are often measured at conditions that are not representative for in situ soil conditions. For example, several types of assays are conducted in pH buffered soil suspensions (sometimes even at pH >9) (Frankenberger and Tabatabai 1991), and because the metal–enzyme interaction is pH-dependent, this might obscure the relationship with effects in the soil. Almost all assays use high substrate concentrations (typically several mM), a condition that is unlikely to occur in situ. Moreover, the colorimetric reaction that is often required in enzymatic assays can also be subject to effects of metals (Nannipieri et al. 1997). Therefore, enzyme assays must be assessed with great care, and they were often not selected for the derivation of soil threshold concentrations in soil risk assessments.

## JURISDICTION-SPECIFIC APPROACHES THAT INCORPORATE MICROBIAL DATA

Many jurisdictions, including Canada, Germany, the Netherlands, the EU, and Australia incorporate soil microorganism assays into their respective screening-level soil assessments. The results of tests with microorganisms have been used together with those for plants and invertebrates to develop soil values at different protection levels (e.g., depending on land-use) (NEPC 2011). Recently, a great deal of research has been invested in developing ecological soil threshold values for metals within the framework of European risk assessments, and detailed protocols were developed to incorporate bioavailability corrections into these ecological soil standards (Smolders et al. 2009). Because the data for the European risk assessment dossiers for Cu, Ni, Zn, and Mo are presented as case studies in this article, the goal and protocols of this European risk assessment are described below.

The European Registration, Evaluation, Authorisation and Restriction of Chemicals (REACH) Regulation (EC No 1907/2006) specifies that industry must demonstrate convincingly that it can produce and use its substances safely. In contrast to regulations that deal with soil quality assessments and specify screening-level soil concentrations (e.g., Eco-SSLs in USEPA [2005]), REACH deals with the toxicological and environmental risks chemicals pose during their entire life-cycle. However, the underlying (eco)toxicity data can be used in both types of regulations. Under REACH, measured or predicted exposure concentrations are compared with a predicted no effect concentrations (PNEC) for each environmental compartment (water, sediment, and soil). The scope of the terrestrial effects assessment under REACH is restricted to a narrow range: to nonvertebrate organisms living the majority of their lifetime within the soil, with exposure routes limited to soil ingestion or direct contact pathways. Three groups of terrestrial organisms are defined under REACH: plants, soil invertebrates, and microorganisms (ECHA 2008a).

The REACH Regulation focuses on the absence of effects, and therefore the toxicity values selected are 10% inhibition effective concentrations (EC10; preferred) and no observed effect concentrations (NOEC) values for chronic endpoints. In many jurisdictions, NOEC and EC10 values were used interchangeably. In this article, we report how microbial data have been used in various jurisdictions. However, it is beyond the scope this article to make recommendations regarding which of these toxicological benchmarks is the most defensible for setting threshold concentrations. For many metals and metalloids, numerous toxicity data (i.e., chronic NOEC or EC10 values for more than 10–15 different species or processes) are available for effects on terrestrial organisms. The PNEC is calculated by dividing the concentration associated with the HC5 (hazardous concentration for 5% of species) of an SSD that includes chronic endpoints for plants, invertebrates, and microorganisms by an assessment factor (between 1 and 5). The size of the assessment factor is determined on a case-by-case basis, taking into account the identified uncertainties (ECHA 2008b).

The selected relevant endpoints for soil microorganisms focus on microbial growth and functional parameters such as respiration, nitrification, and mineralization. The REACH Guidance document lists the following microbial tests: OECD 216, OECD 217, ISO 14238, ISO 14239, ISO 15685, and ISO 17155. Both nonstandard endpoints and nonstandard methods can also be accepted. However, these should be evaluated in relation to ecological relevance, and must be properly identified and characterized to ensure that endpoints are relevant and the methods used and results reported are reliable. The geometric mean of multiple endpoints for a given species or process should be used as the input value for the generation of the SSD to ensure that each species or process is represented only once within the SSD.

Because total metal concentration in soil is not a good predictor of metal bioavailability and the resulting toxicity in soil, bioavailability correction models have been developed for several metals to account for the effects of aging, weathering, time, and soil properties (pH, organic matter content, clay content, etc.) on metal bioavailability and toxicity in soil, and to correct for differences in bioavailability between test conditions in the laboratory compared to site-specific field conditions (Smolders et al. 2009). When appropriate correction models are available, the EC10 or NOEC values are corrected for differences between laboratory and field conditions with the Leaching-Aging Factor (LAF) (Supplemental Data Table S2). The toxicity data are then normalized for differences in bioavailability among soils based on empirical models for microbial endpoints (see Supplemental Data Table S3), according to the procedures outlined in ECHA (2008c) and Smolders et al. (2009). For each species or microbial process tested, the geomean of the normalized data is calculated, and HC5 is derived from the SSD.

## METAL TOXICITY FOR MICROBIAL PROCESSES: DATASETS FOR Cu, Ni, Zn, **AND** Mo

Multiple relevant and reliable data for microbial endpoints were identified for the REACH dossiers for Cu, Ni, Zn, and Mo (Table2). These data are discussed below as case studies for metal toxicity corresponding to microbial endpoints. All toxicity data are expressed as added metal concentrations (i.e., without the natural metal background concentrations for the soils tested). The data used in the effects assessment for the European REACH Regulation were based on organisms and exposure conditions relevant for Europe. Excluding all data derived in non-EU soils would considerably reduce the amount of data to be used; therefore, data based on soils collected outside Europe were also used when corresponding soil properties were within the range representative of Europe. Inclusion or exclusion of enzyme-based data was discussed on a case by case basis for the various metals, based on the quality of the studies and potential methodological concerns (e.g., potential effects of added Cu on some color reactions and spectrophotometric analyses). Therefore, no general rule has been followed regarding inclusion or exclusion of enzyme-based data.

**Table 2:** Summary of data accepted for use in European REACH dossiers for Cu, Ni, Zn, and Mo for toxicity to microorganisms (based on added concentrations; mg/kg)

Metal	Endpoint	Number of data	Range	Geomean generic	Geomean normalized^a^
Cu	Microbial biomass	2	118–468	235	66
Cu	Substrate-induced respiration	26	12–1200	108	100
Cu	Glutamic acid decomposition	3	55–400	107	36
Cu	Plant residue mineralization	18	50–2400	203	288
Cu	Basal respiration	2	150–400	245	290
Cu	Nitrification	22	25–1200	173	271
Cu	N mineralization	2	100–268	164	102
Cu	Ammonification	1	/b	1000	1166
Cu	Denitrification	1	/	100	85
Ni	Nitrification	10	44–439	116	139
Ni	N mineralization	2	20–257	72	220
Ni	Substrate-induced respiration	10	22–376	127	141
Ni	Plant residue mineralization	8	42–446	152	218
Ni	Basal respiration	4	27–2542	299	176
Ni	Glutamate respiration	4	55	55	37
Ni	ATP content	1	/	77	74
Ni	*Aspergillus flavipes* (hyphal growth)	1	/	347	447
Ni	*Aspergillus flavus* (hyphal growth)	1	/	393	507
Ni	*Aspergillus clavatus* (hyphal growth)	1	/	13	17
Ni	*Aspergillus niger* (hyphal growth)	1	/	400	516
Ni	*Penicillium vermiculatum* (hyphal growth)	1	/	102	132
Ni	*Rhizopus stolonifer* (hyphal growth)	1	/	288	371
Ni	*Trichoderma viride* (hyphal growth)	1	/	530	683
Ni	*Gliocladium sp*. (hyphal growth)	1	/	200	258
Ni	*Serratia marcescens* (colony count)	1	/	155	200
Ni	*Proteus vulgaris* (colony count)	1	/	15	19
Ni	*Bacillus cereus* (colony count)	1	/	285	367
Ni	*Nocardia rhodochrous* (colony count)	1	/	177	228
Ni	*Rhodotorula rubra* (colony count)	1	/	247	318
Ni	Urease	5	90–2300	281	257
Ni	Phosphatase	3	251–7021	875	595
Ni	Arylsulfatase	5	272–7080	993	1092
Ni	Dehydrogenase	1	/	8	12
Ni	Saccharase	1	/	77	75
Ni	Protease	1	/	77	75
Zn	Ammonification	1	/	1000	2716
Zn	N mineralization	7	100–446	211	1285
Zn	Denitrification	1	/	39	127
Zn	Nitrification	19	38–424	120	337
Zn	Acetate mineralization	1	/	303	1019
Zn	Glutamic acid mineralization	3	30–100	55	392
Zn	Substrate induced respiration	16	30–1400	204	722
Zn	Basal respiration	9	17–204	83	418
Zn	Plant residue mineralization	11	38–1400	241	686
Zn	Arylsulfatase	4	105–2353	406	1246
Zn	Dehydrogenase	2	76–500	195	1854
Zn	Phosphatase	3	160–623	826	1850
Zn	Urease	4	30–460	73	225
Mo	Nitrification	8	35–3840	603	1210
Mo	Substrate-induced respiration	6	10–1820	160	348
Mo	Plant residue mineralization	4	164–3617	769	3098

a Geometric mean of all data for 1 species or microbial process, after normalization to a reference soil having pH = 6, 1% organic carbon, 10% clay, eCEC = 10 cmol_c_/kg, and background Zn = 50 mg/kg.

b No range presented for studies reporting a single value.

### Copper

In total, 77 NOEC or EC10 values were selected for microbial endpoints for Cu (Table2). The selected NOEC or EC10 values comprise toxicity endpoints for microbial processes (*n* = 77) and microbial biomass (*n* = 2). The parameters for microbial processes are based on the C cycle (basal respiration, substrate induced respiration, plant residue mineralization, and glutamic acid decomposition; *n* = 49) and the N cycle (nitrification, N-mineralization, ammonification, and denitrification; *n* = 26). The biomass parameters were based on the amount of microbial C and N in the soil. Enzymatic parameters were deemed unreliable, thus were not considered in this effects assessment. Selected NOEC or EC10 values (based on added Cu concentrations) ranged from 12 mg/kg dry weight (dw) (substrate induced respiration) to 2400 mg/kg dw (plant residue mineralization).

### Nickel

In total, 68 NOEC or EC10 values were selected to assess Ni toxicity to soil microorganisms (Table2). The selected NOEC or EC10 values included parameters for microbial processes (*n* = 39), microbial species (*n* = 13), and enzymatic processes (*n* = 16). The functional parameters are based on the C cycle (basal respiration, substrate induced respiration, plant residue mineralization, glutamic acid decomposition, and ATP content; *n* = 27) and N cycle (nitrification and N-mineralization; *n* = 12). Enzymatic parameters were further evaluated in the effects assessment, and 6 enzymatic processes were compiled in the database (urease, phosphatase, arylsulfatase, dehydrogenase, saccharase, and protease). NOEC or EC10 values based on added Ni concentrations ranged from 7.9 mg/kg dw (dehydrogenase) to 7080 mg/kg dw (arylsulfatase).

### Zinc

A total 108 individual EC10 or NOEC values from microbial assays were selected (Table2). These values represent 4 N transformation processes, 5 C transformation processes, and 8 enzymatic processes. They range from 178 mg Zn/kg dw for basal respiration to 2623 mg Zn/kg dw for phosphatase. The background Zn concentration was selected as a correction factor for Zn bioavailability and toxicity for microorganisms (see Supplemental Data Table S3). Information on the background Zn concentration, allowing correction for differences in bioavailability among soils, was only available for 76 NOEC or EC10 values, representing 13 microbial processes (4 for N cycle, *n* = 23; 5 for C cycle, *n* = 40; and 4 enzymatic processes, *n *= 13). Because background Zn concentrations are used to normalize toxicity data, only the data with this information available were considered further. The total range in NOEC/EC10 values for the data set with results for background Zn concentration was the same as for the total data set for microorganisms.

### Molybdenum

In total, 18 individual EC10 or NOEC values for 3 microbial processes were selected to assess the terrestrial effects of Mo (Table2). These values represent parameters for microbial processes based on the C cycle (substrate-induced respiration and plant residue mineralization; *n* = 10) and N cycle (nitrification; *n* = 8). Selected NOEC and EC10 values, based on added concentrations, ranged from 10 mg Mo/kg soil dw (substrate induced respiration) to 3840 mg Mo/kg dw (nitrification).

## DISCUSSION

### Variability comparison among toxicity data established for soil processes, plant, and soil invertebrate endpoints

Microbial endpoints tend to be highly responsive to changes in noncontaminant factors (e.g., temperature, moisture, O_2_) and can show strong variations across time and distance. However, this is not unique to microbial activity, because early plant growth or root elongation assays are also strongly affected by environmental conditions such as moisture, nutrient availability, and pH. A comparison of various soil microbial and plant growth assays shows that there is no clear distinction in robustness (i.e., variation of control response across different soils) between microbial and plant assays (Broos et al. 2005). In this study, an overall negative correlation was observed between the sensitivity to metal stress and the robustness of an endpoint, illustrating that in these assays sensitive endpoints are highly variable among control soils.

Microbial endpoints do not show consistently higher variability in individual NOEC and EC10 values (as expressed by the max–min ratio) compared to toxicity values for single-species tests with plants or soil invertebrates, based on the REACH dossiers for Cu, Ni, Zn, and Mo (Table3). Normalized values are not confounded by differences in bioavailability among the soils tested for the various endpoints; therefore, normalization to the same reference soil properties allows comparison of the toxicity endpoint values. No general trend in variability was observed for the metals discussed when microbial endpoints data were compared to data from plants or soil invertebrate tests. Hence, there is no evidence for a higher variability among microbial endpoints compared to those for plants or soil invertebrates.

**Table 3:** Variability and sensitivity of microbial endpoints compared to those for single-species tests for plants and soil invertebrates

			Range (max–min ratio; mg/kg)	
Metal	Organisms	*n*^a^	Original data	Normalized geomean^b^
Cu	Plants	67 (9)	16–660 (41)	56–360 (6)
Cu	Invertebrates	108 (10)	3.2–1390 (434)	55–675 (12)
Cu	Microorganisms	77 (9)	12–2400 (200)	36–1166 (32)
Ni	Plants	68 (11)	10–1101 (110)	39–465 (12)
Ni	Invertebrates	37 (6)	36–1110 (30)	120–893 (7)
Ni	Microorganisms	68 (26)	8–7080 (896)	12–1092 (90)
Zn	Plants	31 (9)	32–5855 (183)	67–1938 (29)
Zn	Invertebrates	61 (8)	15–1634 (112)	222–2477 (11)
Zn	Microorganisms	76 (13)	17–2623 (154)	127–2716 (21)
Mo	Plants	45 (5)	4–3476 (869)	73–207 (3)
Mo	Invertebrates	23 (3)	8–1865 (237)	137–2001 (15)
Mo	Microorganisms	18 (3)	10–3840 (384)	348–3098 (9)

a Number of individual toxicity data; values in parentheses show the number of species or processes.

b Geometric mean of all data for one species or microbial process, after normalization to a reference soil having pH = 6, 1% organic C, 10% clay, eCEC = 10 cmol_c_/kg, and background, Zn = 50 mg/kg.

### Comparison of relative sensitivities to metals among microbial, plant, and soil invertebrate endpoints

**Figure 1:**
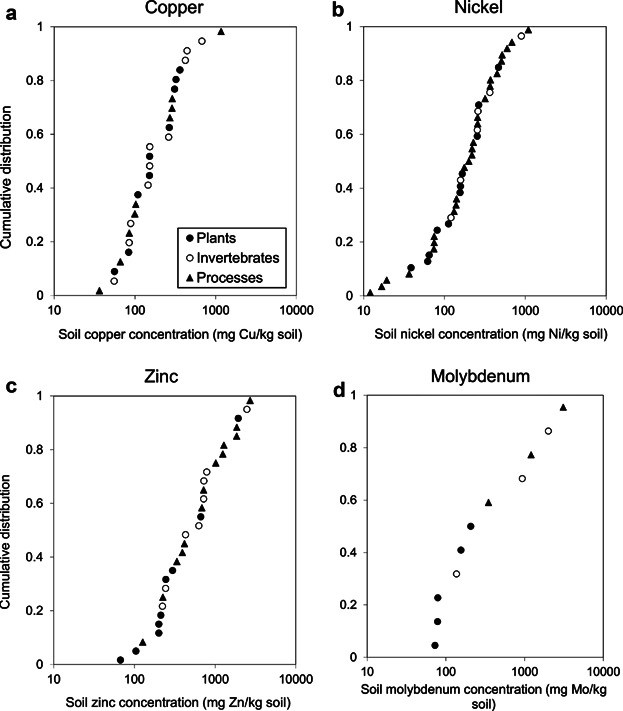
Species sensitivity distributions (SSDs) based on NOEC and EC_10_ values for Cu, Ni, Zn, and Mo.

**Figure 2:**
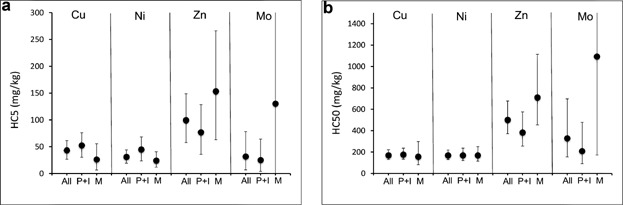
Estimated HC_5_ and HC_50_ values for species sensitivity distributions (SSDs) with and without toxicity data for microbial endpoints. All = standards for combined plant, soil invertebrate, and microbial endpoints; P+I = plant and soil invertebrate only; and M = microbial only. Points are log-normal means based on log-normal distributions; error bars show 5 to 95% confidence intervals (Aldenberg and Jaworska 2000). All data are normalized to a reference soil having pH = 6, 1% organic carbon, 10% clay, eCEC = 10 cmol_c_/kg, and background Zn = 50 mg/kg.

Clear concentration–response relationships were observed in most toxicity tests of microbial processes, allowing the calculation of reliable ECx values. Therefore, it was possible to relate specific microbial activities to indications of adverse and unacceptable loads of contaminants in soil. The sensitivities of microbial endpoints overlap with the sensitivities for tests with plants or soil invertebrates (Table3). There is no species or group (plants, soil invertebrates, or microorganisms) that is consistently most or least sensitive. The microbial endpoints are generally distributed across the range of the combined SSD (Figure 1). Addition of the microbial endpoints to those for plants and soil invertebrates does not significantly affect the resulting HC5 or HC50 values compared to the SSD based on data for only plants and invertebrates (Figure 2); differences in HC50 were generally smaller than differences in HC5. However, inclusion of the microbial data decreased the uncertainty for the estimated HC5 or HC50 values (i.e., smaller confidence intervals), thereby increasing the robustness of the SSD. Moreover, including microbial endpoints into the SSD, or environmental risk assessment in general, makes the assessment more relevant as this allows consideration of critical soil functions with respect to soil fertility and nutrient cycling. These soil functions are essential because they also may affect vegetation, habitat, and soil invertebrate communities. Such effects will not become apparent in standard plant and invertebrate tests because nutrient and food supply is generally optimized in these assays. The examples for Cu, Ni, Zn, and Mo show that protecting higher-order organisms (plants and invertebrates) also protects the microbial communities (i.e., lower HC5 for plants and invertebrates compared with microbial processes) (Figure 2). These comparisons were based on the EC10 and NOEC values only. Data for Mo show that at higher effect levels (e.g., EC50 values), microbes are often less sensitive than are plants (data not shown). This may be attributed to functional redundancy (i.e., changes in community composition will prevent large changes in the microbial endpoints measured), resulting in a less steep dose–response curve compared to single-species tests. However, this phenomenon was not observed for all microbial endpoints; it was much more pronounced for endpoints related to C mineralization in comparison to nitrification assays. Protection of microbial functions and processes is considered more relevant for protection of soil functions and ecosystems than protecting the individual most sensitive species (Cairns 1986). These observations warrant the inclusion of the tests for soil microbial functions into an ecological risk assessment, and in the development of SCVs for metals and metalloids.

### Comparison of relative sensitivities to metals among soil enzyme and other microbial toxicity endpoints

The ecological relevance of enzymatic endpoints for effects assessments of metals and metalloids in soil was questioned during the preparation of the REACH dossiers. Generally, enzyme assays only determine the activity of an individual specific enzyme, whose reaction is frequently a component in a multi-enzymatic process. This enzyme activity in the field might be limited by other factors such as substrate availability or other rate-determining factors. Therefore, the relevance of a single enzyme activity is considered lower than the relevance of net rate measurements of an overall process. Tests focusing on enzyme processes were included in the terrestrial effects assessment for Ni and Zn, but not for Cu and Mo. When compared to toxicity data for other microbial assays or that for plant and invertebrate tests, toxicity data for enzymatic processes showed inconsistent differences in sensitivity (Table4). For Zn, the enzymatic processes were generally less sensitive compared to parameters for microbial processes, whereas the reverse was true for Ni. Including or excluding toxicity data for enzyme assays usually has a marginal impact on the overall SSD and resulting HC5.

**Table 4:** The effect of including or excluding the enzyme assays on the HC5 for Ni and Zn^a^^b^

	Ni		Zn	
Data group	*n*	HC5 (mg/kg)	*n*	HC5 (mg/kg)^b^
Only microbial processes without enzyme assays	52 (20)	30.9 (14.5–51.8)	63 (9)	136 (42.1–256)
Only microbial processes with enzyme assays	68 (26)	24.2 (11.9–40.5)	76 (13)	153 (62.8–266)
All data without enzyme assays	157 (37)	37.4 (23.8–52.8)	155 (26)	93.9 (52.8–142)
All data with enzyme assays	173 (43)	31.0 (19.5–44.3)	168 (30)	99.7 (58.0–149)

a Based on log-normal distributions, confidence intervals calculated (Aldenberg and Jaworska 2000).

b All data are normalized to a reference soil with pH = 6, 1% organic C, 10% clay, eCEC = 10 cmol_c_/kg, and background Zn = 50.

### Bioavailability correction factors for soil processes

The LAFs used in the REACH dossiers to correct for laboratory-to-field differences in toxicity were selected based on a weight-of-evidence approach, taking into account information on changes in toxicity as a function of time for plants, soil invertebrates, and microbial processes. These changes in toxicity over time are attributed to the decrease in metal bioavailability in soil due to aging and weathering reactions (Smolders et al. 2009). Microorganisms can adapt to elevated metal concentrations (Mertens et al. 2007), which may account for the experimental LAF values being greater for microbial endpoints than those for plants or invertebrates. However, LAF values for various groups of terrestrial organisms are not significantly different (Table5). Therefore, an LAF based on toxicity data for plants, soil invertebrates, and microbial endpoints, as derived for the metal REACH dossiers, is considered appropriate.

**Table 5:** Range in observed LAFs for major groups of terrestrial organisms^a^

Metal	Microorganisms	Plants	Invertebrates
Cu	0.5 to >31	0.6 to >6.1	1.5 to 9.2
Ni	2.5 to >63	0.5 to >19	1.3 to 8.8
Zn	>1.8 to >13	0.8 to >11	1.0 to >12
Mo	0.02 to >4.1	0.2 to 25	0.4 to 36

> =  unbounded values, i.e., no toxicity was found in the aged or field contaminated soil; for the calculation of the ratio, the highest concentration measured in the field contaminated or aged soil has been used as unbounded EC10; LAFs  =  leaching-ageing factors.

a Data from REACH dossiers.

Soil parameters used for normalization were selected from the best fits of toxicity data with each soil factor by linear regression analysis, and the slopes of best fitting regressions were used to account for differences in bioavailability among soils (Smolders et al. 2009). Several soil properties were selected for some metals and metalloids, and this relates to the fact that the best-fitting factors differed among the biological endpoints tested. Generally, eCEC was the best predictor of soil toxicity for these metals; eCEC was best correlated across all the endpoints for Ni, whereas clay content, pH, or background metal concentration were among the parameters best correlated with microbial endpoints for Zn and Cu. There were no distinct differences among the trophic groups. Metal toxicities to microbial endpoints are affected by the same soil properties as toxicities to plants or invertebrates. This is important because it indicates that neither additional parameters must be determined, nor additional analytical work required, to include microbial processes data into an SSD for derivation of site-specific SCVs for metals or metalloids.

## RECOMMENDATIONS FOR DERIVING SITE-SPECIFIC SOIL CLEAN-UP VALUES

### Test and endpoint selection

This step is critical as it defines which endpoints are considered relevant for the assessment. Several common, standardized tests have long been used across jurisdictions to assess soil toxicity. These tests have several features in common: 1) they are carried out in natural or artificial soil (i.e., tests using artificial media such as agar plates are rejected), 2) they have well-defined chemical endpoints related to soil functions or processes that are measured as rates, and 3) they are closely linked to key biogeochemical functions that are intrinsically linked to soil fertility. Some examples include potential nitrification rate (ISO 15685), soil respiration (ISO 17155, OECD 217), N mineralization (ISO 14238), and microbial biomass (ISO 14240-1/2). These tests establish specific endpoints that are closely linked to microbial processes.

Both standardized (e.g., ISO 29843-1/2) and nonstandardized genomic tests have been developed to assess microbial diversity. Such tests have been widely used to assess the effects of pollutants; however, they give rise to a wide variety of indices or measures of composition. For example, diversity tests can provide information on species richness, evenness, species diversity estimates, and ordination scores from various multidimensional reductive approaches. Similarity indices can be used to compare how communities are responding to toxicants, thus determining the extent of exposure that causes a microbial community to diverge significantly from control. That microbial community structure is linked to processes is a central tenet of soil microbiology, but proof of in situ community structure linkage to function has proven elusive. At this time, it is not yet clear which numerical estimate of microbial community composition is most relevant for ecosystem functioning, thereby best suited for inclusion in the derivation of ecological soil threshold concentrations, such as site SCV.

The high throughput genomic approaches provide diversity estimates similar to those for the phospholipid fatty acid analysis and also provide exposure–concentration response curves for individual genera. These genera estimates from the current generation of sequencing platforms are believed to be the most quantitatively robust estimates. Thus, genomic approaches can provide an estimate of genera-specific response curves for specific functional guilds (e.g., N fixers, nitrifiers) of microorganisms known to be essential for terrestrial biogeochemical cycles. The results from these genera-based dose–response curves can be readily incorporated into our existing framework. However, it is not clear how to deal with the sheer number of different genera that can be evaluated using these new genomic approaches. Therefore, we suggest that only dose–response curves from N-fixing cyanobacteria and nitrifiers, which are clearly linked to the soil processes of N fixation and nitrification, be included in the derivation of site SCV. However, before including results from these nonstandard tests, further guidance for data selection is needed as outlined below.

### Data selection

The reviewed Australian, EU, and US jurisdictions had clear data selection guidelines (see Checkai et al. this issue) that could be readily adapted to microbial data. We suggest that these guidelines, or similar data quality guidelines, be adopted for studies that aim at the development of site SCV. The selection of an effective concentration (EC) level is based on historical or fairly arbitrary rationale. For example, the Australian approach uses the EC10 and EC30 levels, the USEPA uses an EC20 (preferred level for derivation of Eco-SSL values), the EU uses an EC10, and Canada uses either the EC25 or EC20. There is no scientific evidence demonstrating that the effect level (ECx) for soil microbial processes should differ from those used in other ecotoxicological studies.

### Data treatment: Background metal concentration and bioavailability corrections

Treatment for microbial toxicity data should be the same as for toxicity data from studies with plant and invertebrates (Checkai et al. this issue). In summary, data on the background concentration of metals should be incorporated into the method for deriving SCVs or other site-specific soil threshold concentrations. Recommended SCVs should be expressed in terms of the amount of metal that, if added to the soil, will not exceed the accepted level of toxicity. These concentrations should be calculated by adding the empirical concentration value to the background concentrations specific to the investigated soil.

In case correction models are available for the metal or metalloid assessed, test data should be corrected for differences between laboratory and field conditions (with LAF) and preferentially be normalized to specific soil properties. Predicted SCVs for a soil with low clay, low organic matter content, low eCEC, and low (e.g., 3.3–4.4 for cations but >7 for oxy-anions) pH can be selected to evaluate a generic conservative (i.e., high bioavailability) scenario for metals. Alternatively, site managers can use the available data for soil pH, organic matter, and clay contents, and eCEC from their site, to normalize the toxicity benchmarks to the soil typical of their site, to customize SCV for site-specific soil properties. The normalization procedure for toxicity data for microbial endpoints is similar to that for plants and soil invertebrates described in Smolders et al. (2009). In essence, the correlation of the test response with soil properties (e.g., pH, clay, organic matter, or eCEC) is used to normalize the response to a specific value of these soil properties for the site of interest. This normalization step substantially reduces the variability in bioavailability among the toxicity data for a single endpoint and thereby provides a much more precise estimate of the effect expected for a specific soil. For example, for Cu, Zn, Mo, and Ni, individual data for a single endpoint measured in various soils can typically vary up to 2 or 3 orders of magnitude. After normalization to the same soil conditions, this within-endpoint variation is generally reduced to approximately 1 order of magnitude. Similar reductions in variation after normalizations were observed for toxicity data for plants, soil invertebrates, and microbial processes (Table4).

### SSD development

When sufficient toxicity data are available, as is the case for most metals, SSD should be developed for the derivation of SCVs or similar threshold values. Results from microbial tests can be combined into a single SSD along with the information from soil invertebrate and plant tests (Carlon 2007). Inclusion of data for additional groups of soil organisms or processes increases the ecological relevance and robustness of the SSD. Site managers and regulators can then decide on the specific protection level (HCx) they wish to use to derive a SCV.

We have illustrated this approach with the REACH data sets for Cu, Zn, Ni, and Mo. As evident from the data, this approach produced a precise and plausible SSD. Incorporating the results of the microbial responses did not substantially alter the calculated HC values but did increase the confidence associated with these protection values and provided a statistically more robust determination of an ecological soil standard.

## KNOWLEDGE GAPS

A great deal of knowledge has been generated during the development of the European and Australian soil standards based on soil microbial endpoints. However, a few key knowledge gaps remain, mainly related to determination of relevance of observed effects to some microbial endpoints (e.g., enzymes, community composition). As also discussed above, considerable uncertainty remains on the adequacy of results from some toxicity tests for general soil ecosystem functioning and protection.

1. There is a need for determining which estimates of microbial community composition are linked to results from standardized functional assays. It is not yet clear which types of dimensional reduction or similarity approaches are best suited to predict toxicity. Some similarity measures are best suited to assess treatment groups, whereas others are better at detecting impacts (Kuczynski et al. 2010). A similar informatics analysis is needed to assess which indices provide the most robust estimates of toxicity and how it links to changes in soil microbial processes.
2. The correspondence between the results of massively parallel sequencing and existing toxicity test results needs to be determined. DNA extraction methods have been standardized by ISO 11063 (2010) and 17601 (2012); it is not yet clear how to analyze the resulting data for toxicity evaluations. For example, some work suggests that the variability associated with soil replicates is lower than the overall ecosystem variability (Banerjee et al. 2011); however, differences in primer bias and sensitivity to coextracting PCR inhibitors have made comparisons between soils suspect. The sequencing efforts typically estimate the prevalence of between 130 and 180 different genera (several thousand species) across doses. This then gives rise to 130 to 180 different dose–response curves that must be assessed. Techniques are needed to obtain robust, standardized results for this large number of dose–response curves. The results of these sequencing efforts need to be compared to other common soil toxicity tests to evaluate the plausibility of these responses.
3. Key soil fertility enzyme assays, such as phosphatase and arylsulfatase, are often excluded because they are assumed to measure exposure, not effect. In other words, it is not clear whether a reduction in the enzyme assay indicates an adverse effect in the intact soil ecosystem. Recently, this same concern has been addressed for the PNR: when compared, the results of a PNR assay corresponded well with the results of an isotope dilution activity assessment (Harvey et al. 2012). A similar effort should be undertaken for phosphatase and arylsulfatase enzyme assays using a similar isotope dilution pool approach. This will provide key validation of these 2 enzyme assays, long thought to be key indicators of soil fertility.
4. Although 2 holistic methods assessing overall soil quality have been developed (OECD 56, ISO 18311), research is needed to establish approaches to incorporate these methods into the diagnostic risk assessment paradigm. The litterbag test addresses organic matter decomposition (Römbke et al. 2003), and the bait–lamina test measures the feeding activity of soil organisms (UKEA 2002). Both tests assess key ecological functions of the whole soil community directly in the field, but they require field placement and therefore are sensitive to weather and other site variables that influence biological activity. Research is needed to develop approaches to derive site-specific SCVs for sites where there is not a strong gradient of the chemical of concern.

## CONCLUSIONS

Microorganisms are essential for sustaining soil fertility, soil structure, nutrient cycling, and groundwater purification. Preservation of long-term biogeochemical cycles is key to sustaining the functioning of terrestrial ecosystems. Important to achieving this goal is the need for inclusion of microbial processes in the terrestrial effects assessment for the development of SCVs. The data selected under the REACH Regulation for Cu, Ni, Zn, and Mo showed that microbial processes generally show a similar precision and sensitivity toward metal toxicity compared to plants or invertebrates, based on the ranges of effective concentration values established for microbial processes, plants, and invertebrates.

The high natural spatial and temporal variation of microbial biomass and its potential for functional redundancy and adaptation to elevated contaminant concentrations do not compromise the relevance of the functional endpoints (respiration, mineralization, nitrification, etc.) for ecosystem protection. Supplementing data from plant and invertebrate tests with data for soil processes increases the relevance, robustness, and reliability of the derived threshold value.

Robust protocols for including soil microbial toxicity into the soil effects assessment are available, and bioavailability corrections are generally consistent with protocols and models derived for plants and invertebrates. These protocols can be used as the basis for developing a similar protocol suitable for other jurisdictions (e.g., the United States).

A key step in the incorporation of the microbial data into an environmental risk assessment is a thorough relevance screening of the measured endpoints. Additional research will be required to resolve the remaining knowledge gaps on relevance of microbial tests not directly related to functional endpoints (e.g., microbial community composition endpoints, or high throughput genomic approaches) for terrestrial effects assessments or derivation of SCVs.

## SUPPLEMENTAL DATA

**Table S1.** Ecological functions provided by soil microorganisms and associated assessment endpoints.

**Table S2.** Summary of Leaching-Ageing Factors for Cu, Ni, Zn, and Mo.

**Table S3.** Summary of normalization models selected in the REACH dossiers for microbial endpoints for Cu, Ni, Zn, and Mo (based on added concentrations).

## Supporting Information

All Supplemental Data may be found in the online version of this article.

**Table S1.** Ecological functions provided by soil microorganisms and associated assessment endpoints.^a^ **Table S2.** Summary of Leaching-Ageing Factors for Cu, Ni, Zn, and Mo. ^a^ **Table S3.** Summary of normalization models selected in the REACH dossiers for microbial endpoints for Cu, Ni, Zn, and Mo (based on added concentrations).
