# Supplementary material for: Deriving site-specific soil clean-up values for metals and metalloids: Rationale for including protection of soil microbial processes
Source: Integr Environ Assess Manag. 2014 Feb 26;10(3):388–400. doi: 10.1002/ieam.1513 (PMC4286203; doi:10.1002/ieam.1513)
Supplement: Supplementary file 1 — Table S1. Ecological functions provided by soil microorganisms and associated assessment endpoints.a Table S2. Summary of Leaching-Ageing Factors for Cu, Ni, Zn, and Mo. a Table S3. Summary of normalization models selected in the REACH dossiers for microbial endpoints for Cu, Ni, Zn, and Mo (based on added concentrations). [file ieam0010-0388-sd1.docx]

Table S.1. Ecological functions provided by soil microorganisms and associated assessment endpoints. ^a^

| Soil functions | Assessment endpoints |
| --- | --- |
| Nutrient cycling/Soil fertility | C:N ratio |
|  | Potential nitrification |
|  | Microbial biomass and activity |
|  | Soil respiration rate |
| Functional biodiversity | Nitrifying bacteria |
|  | Carbon sources utilization capacity |
|  | Enzymes |
|  | Nucleic acid microbial population characterization |

^a^ Adapted from Faber and Wensem (2012).

Table S.2. Summary of Leaching-Ageing Factors for Cu, Ni, Zn, and Mo. ^a^

| Metal | Selected LAF |
| --- | --- |
| Cu | 2 |
| Ni | 1+ exp(1.4(soil pH – 7.0)) |
| Zn | 3 |
| Mo | 2 |

^a^ Data from REACH dossiers.

Table S.3. Summary of normalization models selected in the REACH dossiers for microbial endpoints for Cu, Ni, Zn, and Mo (based on added concentrations).

| Metal | Endpoint | Dependent variable | Soil property | Slope | R^2^ | Ref. |
| --- | --- | --- | --- | --- | --- | --- |
| Cu | PNR | log EC50 | log eCEC | 1.09 | 0.64 | Oorts et al. 2006 |
| Cu | SIR | log EC50 | log organic carbon  log Clay | 0.74  0.60 | 0.77 | Oorts et al. 2006 |
| Cu | PRM | log EC20 | log Clay  pH | 0.73  -0.36 | 0.72 | Oorts et al. 2006 |
| Ni | PNR | log EC50 | log eCEC | 0.99 | 0.58 | Oorts et al. 2006 |
| Ni | SIR | log EC50 | log eCEC | 1.33 | 0.92 | Oorts et al. 2006 |
| Ni | PRM | log EC20 | log eCEC | 1.21 | 0.71 | Oorts et al. 2006 |
| Zn | PNR | log EC50 | log background Zn | 0.76 | 0.55 | Smolders et al. 2003 |
| Zn | SIR | log EC50 | log background Zn | 0.76 | 0.42 | Smolders et al. 2003 |
| Mo | SIN | log EC50 | log clay | 1.18 | 0.69 | Oorts 2012 |
| Mo | SIR | log EC50 | log clay | 0.73 | 0.90 | Oorts 2012 |

PNR, potential nitrification rate; SIR, substrate induced respiration; PRM, plan residue mineralization; SIN, substrate induced nitrification.
